# Patients’ Perceptions of Stress Urinary Incontinence Treatment: A Scoping Review of Qualitative Studies

**DOI:** 10.1007/s00192-025-06061-w

**Published:** 2025-02-11

**Authors:** Nienke J. E. Osse, Marian K. Engberts, Hugo W. F. van Eijndhoven, Paul L. P. Brand, Marco H. Blanker

**Affiliations:** 1 Department of Primary- and Long-term Care, University Medical Center Groningen, Hanzeplein 1, 9713 GZ Groningen, The Netherlands; 2 Department of Obstetrics and Gynaecology, Isala Hospital, Dokter van Heesweg 2, 8025 AB Zwolle, The Netherlands; 3 Department of Medical Education and Faculty Development, Isala Hospital, Dokter van Heesweg 2, 8025 AB Zwolle, The Netherlands; 4 Wenckebach Institute for Medical Education, University Medical Center Groningen, Hanzeplein 1, 9713 GZ Groningen, The Netherlands

**Keywords:** Patient perceptions, Qualitative research, Shared decision making, Stress urinary incontinence, Treatment decision

## Abstract

**Introduction and Hypothesis:**

Treatment options for female stress urinary incontinence (SUI) are often offered in a stepped-care approach. However, the shift towards patient-centred care and shared decision making (SDM) has prompted an increased interest in patients’ perceptions of treatment decision making. This scoping review maps the available qualitative research on women’s perceptions of the treatment decision-making process for SUI and identifies knowledge gaps.

**Methods:**

This scoping review was performed according to the Preferred Reporting Items for Systematic review and Meta-Analysis guidelines. Three databases were searched using a systematic search strategy, without restriction in publication date or language. After thorough screening, 19 of the initial 3,473 publications were included.

**Results:**

Four themes were identified; pre-existing experiences and notions that women bring to the consultations (things women consider before their consultation); treatment and patient characteristics (treatment aspects and personal values patients deem important); aspects of the consulting health care professional and facilities (availability of treatment options and counselling styles of physicians); ways of reaching a decision (three different ways that women used to make their decision. There were gaps in the literature on aspects affecting women’s treatment preferences, their preferred decision-making style and how they want to be involved in this decision-making process.

**Conclusions:**

This scoping review provides a global overview of women’s perceptions on and preferences for treatment for SUI, and highlights a lack of knowledge on women’s ideas of the treatment decision process. To provide clinicians with better guidance for their counselling and decision-making approaches, studies on women’s perceptions of the decision-making process and the different decision-making styles are needed.

**Supplementary Information:**

The online version contains supplementary material available at 10.1007/s00192-025-06061-w

## Introduction

There are many different available treatment options for stress urinary incontinence (SUI), ranging from expectant management to surgery. They are often offered in a stepped-care approach. Each of these treatments has been studied extensively and the evidence has been summarised in systematic reviews [1–4]. Conservative treatment includes lifestyle advice, pelvic floor muscle therapy (PFMT) and mechanical devices, such as disposable tampons [3]. When such first-line treatment fails, more invasive therapy is considered [5]. The gold standard for surgical therapy is the mid-urethral sling (MUS). Other surgical options include colposuspension, the autologous fascia sling made from the fascia of the musculus rectus abdominis, or other implantable devices such as the adjustable continence therapy balloon and artificial sphincter [1, 6, 7]. Owing to concerns regarding mesh procedures and a desire to explore minimally invasive treatment options, renewed interest is sparked in the injection of bulking agents [4, 8].

The present guidelines on assessment and treatment in general are predominantly based on efficacy, safety research and cost-effectiveness [9, 10]. Over the last decade, however, medical care, including that for women with SUI, has shifted towards a more patient-centred approach and shared decision making (SDM). This has increased interest in patients’ perceptions of desired outcomes and decision-making processes [11]. Other decision-making approaches are informative decision making, in which the patient decides based on information provided by the physician, and paternalistic decision making, in which the physician decides based on their medical knowledge. SDM is currently the advocated way of making for preference-sensitive decisions with two or more valid treatment options, because of increased patient satisfaction, better informed patients and greater treatment adherence [12–15]. With the increasing availability of surgical and minimally invasive treatment options for SUI, it is becoming more important to understand women’s perspectives on the condition, as its effect on their lives affects their choice of SUI treatment.

Three systematic reviews have examined patient perspectives on SUI, focusing on the impact of the disorder and the effect of MUS surgery on their daily lives [16–18]. The main findings of these reviews were that SUI places a significant burden on the daily lives of women, with patients’ perceptions of SUI depending on lifestyle and cultural and social background [16, 18]. Although experiences of SUI and management strategies were similar across racial and ethnic groups, there were certain populations with different perceptions of their SUI [18]. Non-white women perceived SUI as a consequence of prior experiences, expressing more self-blame for the symptoms than white women; Hispanic women were more secretive about their symptoms, even towards close family members; and Muslim women had difficulty with adhering to the cleanliness requirements for religious obligations [18]. Regarding MUS surgery, patients felt that there was a lack of communication about possible adverse events and the impact of MUS surgery on their social, professional and personal lives [17]. The authors concluded that there was a gap in the literature on patients’ perceptions of treatment and their weighing of multiple treatment options [16]. The aforementioned reviews only slightly touched upon patients’ perceptions of the treatment decision-making process. It appears that more information about this is needed to support SDM in SUI.

### Objectives

This scoping review is aimed at mapping the available qualitative research on patients’ perceptions on the treatment decision-making process for SUI in women and to identify and analyse knowledge gaps in the literature on patients’ perceptions regarding treatment decisions and decision-making styles.

## Materials and Methods

We performed a scoping review following the Preferred Reporting Items for Systematic review and Meta-Analysis guidelines for qualitative reviews [19–21].

### Eligibility Criteria

We included original studies aimed at exploring patients’ perceptions (i.e. any thoughts, views or experiences) on the choice of treatment of SUI in women, in which a qualitative approach was used. We excluded publications without a full-text version and publications lacking qualitative descriptive data. We did not restrict the date or language of the publication. The review protocol was not deposited in an online register.

### Information Sources and Literature Search

On 10 August 2022, the MEDLINE (PubMed), CINAHL and EMBASE databases were searched [22, 23]. We used broad search terms on methodology (“qualitative”, “findings” or “interview”) in combination with the different treatment options, “stress urinary incontinence”, and “females” to identify all relevant papers using a sensitive search approach. The full search strategy can be found in Appendix 1. The reference lists of full-text retrieved publications were also screened to identify additional papers. On 17 April 2024, the search was updated to identify additional recent papers that were published during the review process.

### Selection of Sources of Evidence

All citations were imported into Endnote 20 (Clarivate™, version 20.6, build 17174). After the removal of duplicate citations, the title and abstract of all citations were evaluated by the first author (NO) and double screened by one of three other researchers (MHB, MKE, HvE). If no abstract was available, the full text was reviewed [22]. If one or both researchers included a publication based on title and abstract, its full text was assessed for eligibility. In the case of insufficient or unclear information in a paper, an attempt was made to contact the author. If there was no reply within a month, the article was excluded. Differences between the two researchers in their inclusion of relevant studies were resolved by discussion and consensus.

### Data Charting Process

All relevant information was extracted using a data extraction tool in Google Forms, developed for the purpose of this review and piloted on two papers, after which it was modified as required. The full extraction details and definition of extracted data can be found in Appendix 2 [24].

### Data Items

Two researchers (NJEO, MKE) independently extracted the following data in duplicate: bibliographic information; country and setting; type of incontinence; treatment studied; sample size; patient characteristics; study design and relevance to the review; qualitative analysis method; and the order of constructs. An exact copy of the text was extracted for all relevant themes described in the study [11]. The mentioned strengths, limitations and implications for future research were also extracted.

### Synthesis of Results

In this synthesis, we discuss all themes and subthemes in the context of the publications, illustrated by representative participant quotes from the publications. When available, participants’ age is mentioned with the quote. The extracted data were analysed with inductive thematic analysis, supported by the use of ATLAS.ti 23.4.0 for Windows [25]. The exact copy of the text was open coded by one researcher (NJEO). These codes were discussed with all researchers and adapted until consensus was reached. Two researchers (MKE, NJEO) identified themes and subthemes. The themes and subthemes were also discussed within the research team until consensus was reached.

### Critical Appraisal of Individual Sources of Evidence

Following recommendations for scoping reviews, a critical appraisal of the study validity of individual papers was not conducted [19].

## Results

### Selection of Sources of Evidence

**Fig. 1:**
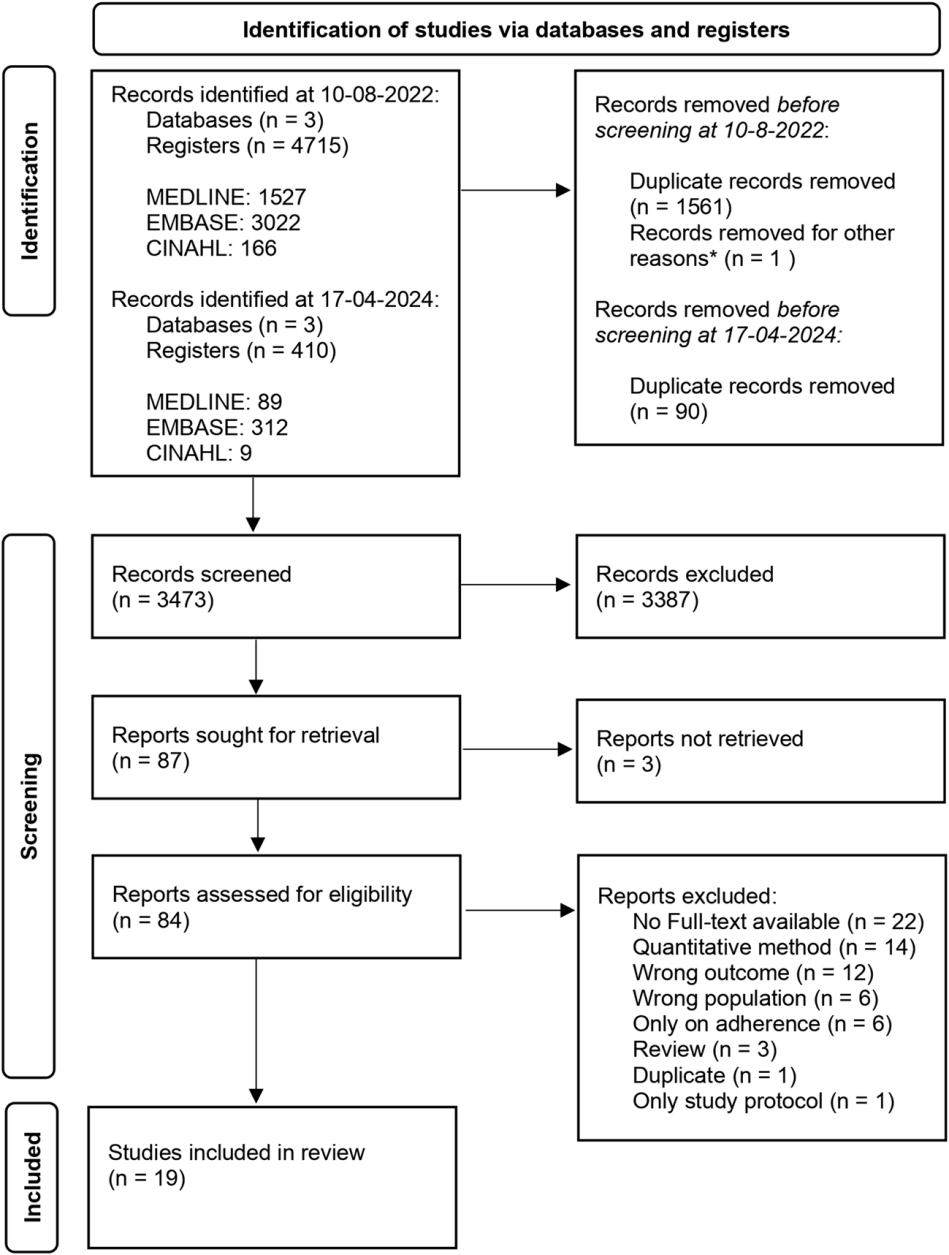
Preferred Reporting Items for Systematic review and Meta-Analysis 2020 flow diagram of the study selection process

We included 19 of the 3,473 unique publications initially identified (Fig. 1). All of the reviewed full-text publications were published in English. Table 1 shows the details of the studies included. We found 10 studies on PFMT [26–31], 4 of which focused on an E-Health approach to PFMT [32–35]. Seven studies focused on surgical management [36–41], 1 of which specifically in patients who suffered from recurrent SUI [42]. One study focused on pessary use [43], and 1 on treatment in general [44]. All studies were published between 2001 and 2023. Most studies were conducted in Europe (*n* = 17), and most were performed in a primary (*n* = 9) and/or secondary care (*n* = 11) setting. Sample sizes ranged from 6 to 42 for studies with a qualitative primary outcome, and from 11 to 212 for studies with a qualitative outcome as a secondary aim. One study did not provide direct quotations from patients [36]. The main themes presented in the studies are displayed in Table 2.

**Table 1:** Details of the studies included

Reference	Country of origin study	Setting	Sample size	Age of patients, mean (SD or range)	Ethnicity of patients	Type of incontinence	Previous treatments	Treatment studied	Qualitative outcome as primary or secondary aim	Study design	Data collection method	Analysis method	Order of constructs
Asklund et al. [32]	Sweden	Primary care	15	47 (27–72)	Not reported	SUI	Not reported	App for PFMT	Primary aim	Mixed methods design	Interviews	Grounded theory	Both order constructs
Basu et al. [44]	UK	Secondary care	16	54 (48–70)	Not reported	SUI	None	Non-surgical and surgical treatments for SUI and/or prolapse	Primary aim	Mixed methods design	Interviews	Grounded theory	Both order constructs
Casteleijn et al. [37]	The Netherlands	Tertiary care	20	49 (23–88)	White, Hispanic, Asian	SUI	None, PFMT, unknown	MUS and bulking agent	Primary aim	Qualitative design	Interviews	Thematic analysis	Both order constructs
Casteleijn et al. [36]	The Netherlands, South Africa	Secondary care	105	52 (± 13.4)	Not reported	SUI	None, PFMT, TVT/TOT, SIMS, bulking agent, pessary	MUS and bulking agent	Secondary aim	Mixed methods design	Interviews	Descriptive analysis	Second order constructs
Dwyer et al. [38]	UK	Secondary care, tertiary care	212	50 (27–84)	Not reported	SUI	Not reported	Surgical treatments for SUI	Secondary aim	Mixed methods design	Questionnaires	Thematic analysis	Both order constructs
Firet et al. [33]	The Netherlands	Primary care	13	(40 to >80)	Not reported	SUI	PFMT	App for PFMT	Primary aim	Qualitative design	Interviews	Grounded theory	Both order constructs
FitzGerald et al. [39]	USA	Secondary care	48	57 (33–82)	Not reported	SUI	Not reported	MUS and autologous fascia sling	Secondary aim	Mixed methods design	Reported as "asked"	Not reported	Both order constructs
Griffiths et al. [26]	UK	Primary care	22	(30–74)	White, Asian	MUI	Not reported	Group and individual PFMT	Primary aim	Qualitative design	Interviews	Thematic analysis	Both order constructs
Hagen et al. [27]	UK	Primary care, secondary care, tertiary care	40	(20–76)	Not reported	MUI	Not reported	Bio-feedback and regular PFMT	Secondary aim	Mixed methods design	Interviews	Framework analysis	Both order constructs
Li et al. [28]	China	Secondary care	22	(25–39)	Not reported	SUI	Not reported	PFMT	Primary aim	Qualitative design	Interviews	Thematic analysis	Both order constructs
Lynch et al. [40]	UK	Secondary care	28	(26–74)	Not reported	MUI	Not reported	Surgical treatments for SUI	Primary aim	Qualitative design	Interviews	Constant comparative method	Both order constructs
Mason et al. [29]	UK	Secondary care	42	31 (21–45)	White, Asian, Black/African American	SUI	Not reported	PFMT in pregnant patients	Primary aim	Mixed methods design	Interviews	Content and inductive analysis	Both order constructs
Nissen et al. [43]	Tanzania	Secondary care	15	55.3 (45–72)	Black/African American	SUI	Not reported	Pessary use for SUI	Primary aim	Qualitative design	Interviews	Qualitative content analysis	Both order constructs
Reynolds and Wilson [30]	Ireland	Primary care	6	(32–45)	Not reported	SUI	Not reported	PFMT in pregnant patients	Primary aim	Qualitative design	Interviews	Thematic analysis	Both order constructs
Sawettikamporn et al. [31]	Thailand	Tertiary care	7	53.2 (40–70)	Not reported	SUI	Not reported	PFMT	Primary aim	Qualitative design	Interviews	Thematic analysis	Both order constructs
Tincello et al. [42]	UK	Secondary care	11	Not reported	Not reported	SUI	Failed surgical treatment	Surgical treatments for recurrent SUI	Secondary aim	Mixed methods design	Interviews	Constant comparative method	Both order constructs
Uberoi et al. [41]	USA	Tertiary care	11	61 (39–88)	White, Hispanic, Asian	SUI	Not reported	MUS surgery	Primary aim	Qualitative design	Interviews, focus groups	Deductive and inductive content analysis	Both order constructs
Wessels et al. [35]	The Netherlands	Primary care	17	(35–78)	Not reported	MUI	None, PFMT	App for PFMT	Primary aim	Mixed methods design	Interviews	Inductive approach	Both order constructs
Wessels et al. [34]	The Netherlands	Primary care	9	(32–68)	Not reported	MUI	None, PFMT, medication for UUI	App for PFMT	Primary aim	Qualitative design	Interviews	Thematic analysis	Both order constructs

*MUS* mid-urethral sling, *MUI* mixed urinary incontinence, *PFMT* pelvic floor muscle therapy, *SUI* stress urinary incontinence, *UUI* urge urinary incontinence

**Table 2:** Main themes found as published by the authors of the studies included

Reference	Treatment studied	Main themes
Asklund et al. [32]	App for PFMT	Enabling my independence
Basu et al. [44]	Non-surgical and surgical treatments for SUI and/or prolapse	Effect of symptoms on quality of life; wish for a permanent solution; success rates of treatment; complications of surgery; confusion about surgical options for prolapse
Casteleijn et al. [37]	MUS and bulking agent	Personal factors; procedural factors; professional factors; social factors; external factors
Casteleijn et al. [36]	MUS and bulking agent	Motivations behind treatment preference for MUS surgery: fear of silicones, one-off procedure, unfamiliarity of bulking agent treatment; motivations behind treatment preference for bulking agent: minimal invasiveness, local analgesia, quick recovery
Dwyer et al. [38]	Surgical treatments for SUI	Invasiveness of the procedure; chance of success; duration of recovery; risk of complications; use of mesh; influence of the clinician; the media; hierarchy of treatments; type of anaesthetic
Firet et al. [33]	App for PFMT	Need to meet; eHealth as a tool to bridge obstacles
FitzGerald et al. [39]	MUS and autologous fascia sling	Newer procedures are better in general
Griffiths et al. [26]	Group and individual PFMT	Embarrassment; expectations and concerns about group sessions before attending; the experience of the group session
Hagen et al. [27]	Biofeedback and regular PFMT	Facilitators of adherence during the active treatment phase; barriers of adherence during the active treatment phase; facilitators of adherence during the maintenance phase; barriers of adherence during the maintenance phase
Li et al. [28]	PFMT	Motivators for receiving PFMT; perceptions of psychosocial burdens while receiving PFMT; modes of decision making
Lynch et al. [40]	Surgical treatments for SUI	How bad is my condition; competing demands; impact of embarrassment and shame
Mason et al. [29]	PFMT in pregnant patients	Performing PFMT during pregnancy; performing PFMT after childbirth; motivation for exercise
Nissen et al. [43]	Pessary use for SUI	Motivation; perceived benefits; perceived barriers
Reynolds and Wilson [30]	PFMT in pregnant patients	Lack of understanding; education
Sawettikamporn et al. [31]	PFMT	Perceptions of SUI; barriers to PFMT; exercise motivators
Tincello et al. [42]	Surgical treatments for recurrent SUI	Limited options due to past treatment experiences and/or personal preferences
Uberoi et al. [41]	MUS surgery	Negative impact on quality of life; physician inquiry/prompting; barriers to care; goals and expectations; varied understanding of surgery; outcomes; outcomes beyond SUI
Wessels et al. [35]	App for PFMT	Accessibility; awareness; usability; adherence
Wessels et al. [34]	App for PFMT	Personal factors; app factors; awareness; adherence

*MUS* mid-urethral sling, *PFMT* pelvic floor muscle therapy, *SUI* stress urinary incontinence

### Synthesis of Results

**Fig. 2:**
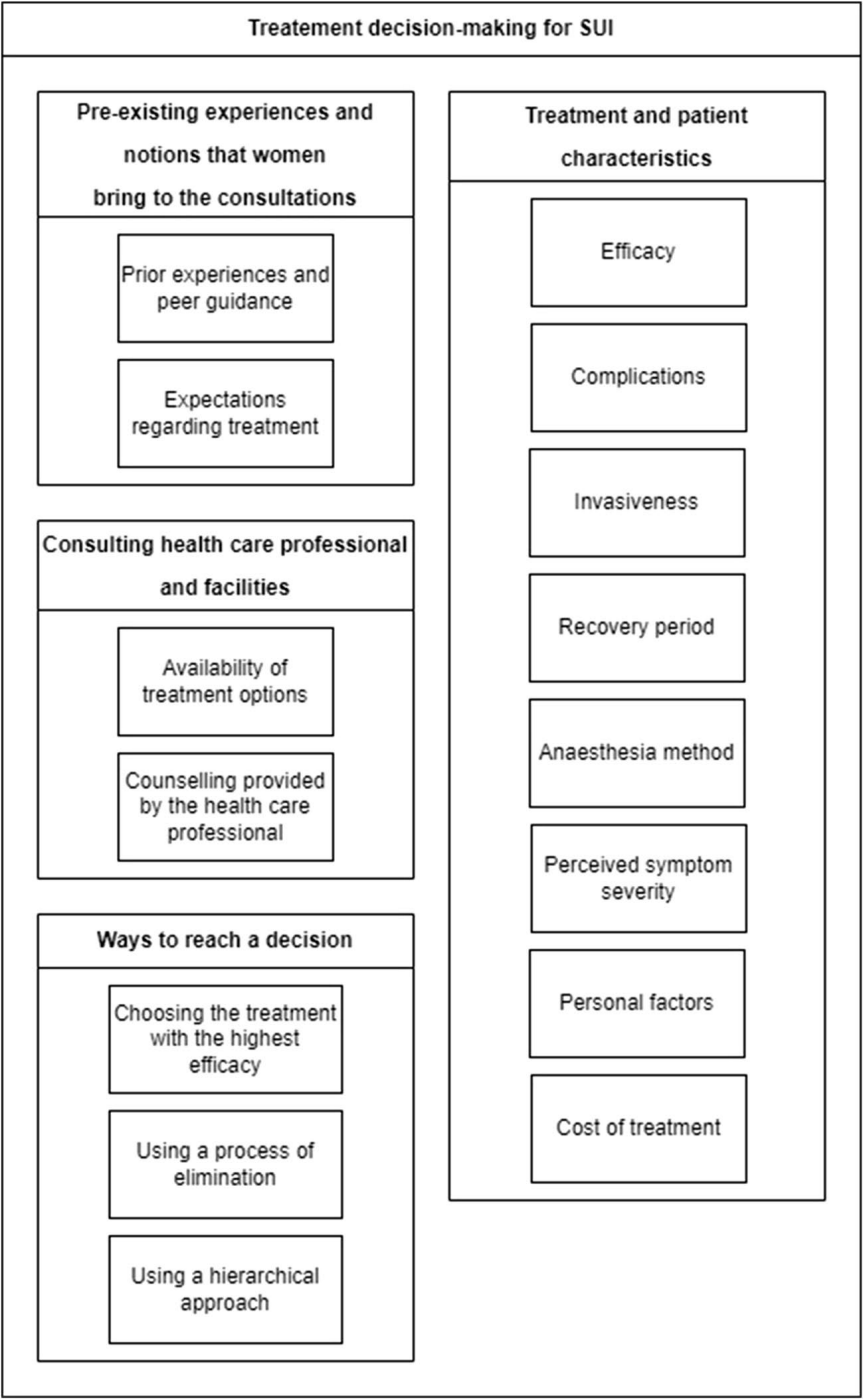
Themes and subthemes identified in the treatment decision-making process for stress urinary incontinence (SUI)

Across all studies, four main themes were identified in the treatment decision-making process of patients with SUI. “Pre-existing experiences and notions that women bring to the consultations” about the things that women consider before their consultation, “treatment and patient characteristics” about the treatment aspects and personal values that patients deem important when making a treatment decision, “aspects of the consulting health care professional and facilities” about the availability of treatment options and the counselling styles of their physician, and “ways of reaching a decision” about the three different ways in which women make their treatment decision. Each theme consisted of multiple subthemes (Fig. 2).

### Pre-Existing Experiences and Notions that Women Bring to the Consultations

The first theme involved two underlying aspects: “prior experiences and peer guidance” and “expectations regarding treatment”.

#### Prior Experiences and Peer Guidance

Multiple studies showed that women take their own previous experiences regarding treatment into account when deciding on a treatment [26–31, 33, 34, 36–38, 40–43]. A UK study quoted a woman who discussed the possibility of repeating a surgery [42].

“I just wanted something that I knew was going to work. I never considered it [another option], I just wanted something, because I’d had it done before…I knew it was going to last me for the rest of my life.”

Women also received peer guidance and experiences from friends and family when considering treatment options [26, 28–31, 37, 38, 40]. A UK study illustrated this with a woman deciding to start PFMT during her pregnancy because she saw the suffering of those around her [29].

A Chinese study quoted a woman who decided on a specific treatment centre, because it was recommended by a friend [28].

My friend recommended me to come here. She said that the postpartum rehabilitation here was very good. She improved a lot after she did PFMT here.” (29 years)

Some women heard stories regarding treatment options in the media. Especially in the UK, the MUS has had negative media coverage [38].

“I didn’t want the mesh due to press”.

Women also had pre-existing knowledge on specific treatment options or considered the lack thereof in decision making [28–30, 33, 36, 37, 41, 43]. A Dutch study on preferences between MUS and bulking agents quoted a woman on the unfamiliarity of bulking agents [36].

“I think the bulk injections are scary…I do not know, maybe because you have heard little about it”.

A Tanzanian study on pessary use found that misinformation on treatment can highly influence treatment decision making [43].

“As for me I have accepted it, but other women need more information about this because most think this instrument [ed. pessary] is cancerous. […]. Due to fear, yes, they prefer using pieces of cloth than this [ed. pessary]. They need more education about this and as for me I didn’t have a problem accepting it”.

#### Expectations Regarding Treatment

Women reported having pre-existing expectations regarding the treatment for their SUI, with treatment goals ranging from prevention to cure [27–29, 31, 35, 37, 40, 41]. In a Dutch study, there were women who only sought improvement of their SUI complaints [37].

“I am not like: I should be dry until the last drop. That is not a goal for me…So, then I still prefer the bulk injection, because when you have improvement [refers to symptoms of SUI] it can be acceptable for everyday life.”

Other women wanted to solve all their pelvic floor issues with their incontinence treatment, as shown in a US study on MUS surgery [41].

“Have things back to normal... to be able to know when I had to urinate... to have bowel issues resolved.”

A Chinese study illustrated this with a woman whose expectations of the recovery time influenced her treatment decision [28].

A UK study on group PFMT found more treatment-specific expectations that influenced the decision to start treatment [26].

“(I) had pre-conceived ideas of a workshop atmosphere, that it would be loads of little exercise mats on the floor, all lying down in positions in leotards.” (30 years)

The wording used for treatment options also seemed to have an impact on patients’ expectations of treatment outcomes, as shown by a US study [39]. Women showed strong pre-existing beliefs regarding “new treatment options”, both positive and negative.

“I have to assume it is better or it would not have been approved for use”.

### Treatment and Patient Characteristics

This theme comprised eight aspects that patients take into consideration when choosing an SUI treatment: “efficacy”, “complications”, “invasiveness”, “recovery period”, “anaesthesia”, “symptom severity”, “patient factors” and “cost of treatment”.

#### Efficacy

Multiple studies from different countries showed that most women deemed efficacy to be the most important issue in decision making [36–39, 43, 44], as illustrated in a Dutch study [37].

“The most important thing for me is it must be efficacious”. (48 years)

Considering the duration of the treatment's effect, many women experienced only temporary relief from PFMT exercises and felt that it was insufficient to cure their symptoms [43, 44].

A Dutch study [37] illustrated that some women based their treatment decisions not only on efficacy but also on the amount of experience with the treatment and the fact that long-term results are available [36, 37].

In a UK study, participants explicitly mentioned that they deemed efficacy to be more important than the risks of treatment [38].

“Efficacy/outcome balanced with acceptable risks. Lots of media scare stories about mesh/tape but on reading NICE guidance and the few research documents I could Google, I felt on balance it was a better option long term”.

#### Complications

In most studies on surgical treatments, women defined complications and risks very vaguely [36–38, 44]. Women were more inclined to choose a treatment with low risks, as in a UK study [38].

However, some women specifically named pain and post-voiding dysfunction as potential risks [37, 38]. A woman in a Dutch study weighed pain, recovery time and the risk of needing another surgery when deciding on her treatment [37].

More women weighed their perceived risks against the possible efficacy, as illustrated in a UK study [38].

Some women were concerned about the material that bulking agents and MUS are made of [36–38]. Some women even considered avoiding the use of a mesh to be more important than the efficacy of a treatment. A Dutch woman was concerned about the components of the gel that the bulking agent she was offered was made of [37], and a British woman was positively inclined towards using her natural body tissues [38].

#### Invasiveness

Overall, women preferred to avoid surgery if they had the option [27, 31, 37], as illustrated in a Thai study [31].

“… if I’m not getting better… may need surgery… which I really don’t want to have”. (60–70 years)

If they had to choose between surgeries, they preferred a minimally invasive treatment [36–38], as described in a Dutch study [37].

Women also expressed fear about the surgical approach in a UK study [40].

“I didn’t think they would actually be doing it through the vagina. So, obviously anything to do with your private area you’re going to be a bit nervous about. It’s something completely different that, the only person that’s ever been there is when I’ve had children”. (26 years)

#### Recovery Method

Recovery time was often taken into account when considering treatment options [28, 36–38, 43].

Women mostly considered recovery time if they feared it would interfere with their personal or professional life [38]. When comparing conservative therapy with surgery, recovery time had a major influence, as demonstrated in a Tanzanian study [43].

“It’s good [the pessary] because if you explain your problem to the doctors, they can advise you in a simple way compared with an operation. Operation will first disturb you, take your time and it will give you pain”.

#### Anaesthesia Method

Women’s views on type of anaesthesia ranged from not wanting to preferring general anaesthesia, and included women who did not take the type of anaesthesia into account when making a decision regarding their treatment [36–38, 42].

A British woman described her view of general anaesthesia as the following [38];

“Don’t like being put to sleep as I am scared”.

#### Perceived Symptom Severity

Women weighed the options for treatment against their perceived severity of symptoms, when they deliberated whether their symptoms were bad enough to warrant surgery [28, 37, 40], as described in a UK study [40].

“…my problem doesn’t feel bad enough to go for surgery…I really wanted to keep going with the physio and try and sort it out that way…”. (51 years)

#### Personal Factors

Women tried to choose a treatment that would interfere as little as possible with their personal lives [28, 38, 40], as demonstrated by a British woman [38].

“I have two toddlers to look after so having an operation was not an option for me at this time. I am a working mum running my own business. My husband works full time. The treatment I have picked is best as I would only be off work for a day”.

Women also considered their general health status, including the possible future wish for pregnancy [38, 40]. Some women also took their age into account when making a treatment decision for their SUI [28, 37], as demonstrated in a Chinese study [28].

“The older generation like my mother said that some people (peers) around her needed to wear adult diapers. No one in their era had ever received treatment. I am afraid that the leakage of urine will become more serious when I get old, so I must do it”. (27 years)

#### Cost of Treatment

Costs were mentioned occasionally as a treatment decision-making factor [28, 37], illustrated by a Chinese woman who had to reduce the number of PMFT appointments [28].

#### Consulting Health Care Professional and Facilities

There were two aspects of the consulting health care professional and facilities: “availability of treatment options” and “counselling provided by the health care professional”.

#### Availability of Treatment Options

In developing countries such as Tanzania, the availability of treatment options is limited [43].

“For sure it’s good [the pessary] and if these white people [the researchers] could stay here and visit us in our villages it could be better because if you tell a woman to come all the way to here [to the hospital] she will tell you she doesn’t have time or money. But if they come to those dispensaries near our village these women will be educated”.

When women had access to health care, they deemed it important that they were able to build a rapport with the health care professional [26–28], as described by a British woman [27].

“And it’s very motivating… you know, seeing someone who’s interested in you, who wants to help you is terribly motivating… ‘cause otherwise you’re just on your own, ‘cause you don’t chat to your friends about it… the only person I’ve ever really spoken to about this [UI] is [OPAL therapist] and the nurse specialist”.

When considering different types of PFMT, some women preferred to have physical contact with a therapist [28, 33, 37, 38], whereas others preferred to do exercises in their own home [27, 29, 32–34].

A participant in a Dutch study explained her preference for being able to consult a health care professional [33].

“Pelvic floor muscle exercises are pretty tough. […] It’s easy to pick the wrong muscles although you might be thinking you’re doing well. It would be nice to have an expert to check it; the computer cannot do that”. (52 years)

A Swedish woman explained her preference for doing her exercises at home [32];

“That was what was so good about this, I can do this at home myself, no need to book an appointment, find the time and suit others”.

When considering surgical treatment, the ease of treatment also had an influence. Some women preferred bulking agent injections as they could be done in an outpatient setting without hospital admission, whereas others preferred surgery over conservative management as they would not have to come for repeated clinic visits [36, 37, 44]. Transport and parking problems also played a factor in choosing a treatment [27].

### Counselling Provided by the Health Care Professional

Some women felt that they were only offered limited options, whereas they wanted to be informed of more options [38, 41, 42], as stated in a US study [41].

The explanation of the options could also be unclear to women, as explained in an American study demonstrating a lack of understanding of the treatment options [41].

Some women reported that they highly valued the health care professional’s advice in the decision-making process [28, 37, 38, 43]. A Tanzanian woman stated her trust in her doctor as follows [43].

“Something that motivated me to use the pessary was because the doctor advised me to use it and also I had some fear that if I won’t use [ed. it] the problem might get worse and I normally obey the orders given by doctors so that the problem doesn’t get worse”.

By contrast, a Chinese woman stated that she valued having autonomy in making a decision [28].

“Today, the doctor prescribed electromyographic biofeedback to me, but I didn’t think I needed it. I thought I could do it myself at home, I just wanted to do the manual therapy, I wanted to cancel the electromyographic biofeedback. Then, the doctor heard my opinion”. (28 years)

### Ways of Reaching a Decision

We identified three different ways in which women make a treatment decision: choosing the treatment with the highest efficacy, using a process of elimination and using a hierarchical approach (Fig. 2).

#### Choosing the Treatment with the Greatest Efficacy

The importance of efficacy was also displayed in the way in which patients made decisions, for instance, some women chose the treatment option with the highest efficacy [36–38, 41, 44]. By doing so, they hoped for a one-off treatment, as related in a UK study [44].

“I just want to come in and they say, that’s that, you’re sorted. If I’m going to get something done, I want to have something that is most likely to work—even if it’s a bit more inconvenient”.

#### Using a Process of Elimination

For other women, however, success rates were not the most important in making a decision. These women viewed the treatment decision making for SUI as a process of elimination, in which they crossed out the options that were unfit, owing to their comorbidities, or that they had already tried, as demonstrated in a UK study on recurrent SUI [42].

“I felt as though everything else had failed… and, you know, I thought well, I’ll try this [colposuspension] I didn’t know what to try next really […] I sort of jumped at it, you know, wholeheartedly, think “ooh, is it going to work? Hopefully it will”.

#### Using a Hierarchical Approach

Finally, there were women who preferred a hierarchical approach to their decision making [27, 28, 31–38]. They first wanted to try the least invasive treatment option with the lowest risk, such as PFMT on an e-Health platform, where the threshold for starting treatment could be lower. Even though this Swedish woman did not have high expectations of the outcome of PFMT on an e-Health platform, she wanted to give it a try first [32].

“I probably expected that after three months I would be able to feel like I had done everything I could. I did not expect to feel like, oh, now I’m going to be cured for the rest of my life, but to know that I had at least given it an honest chance.”

If that proved to be insufficient, women were willing to try bulking agent injections, which they deemed to be minimally invasive. Especially because, as demonstrated by a British woman, this treatment did not interfere with the possibility of future, more invasive treatments [38].

“I prefer to try least invasive method before I would even consider any other. I was told that other alternatives would not be affected by having the least invasive treatment”.

## Discussion

### Summary of Evidence

From 19 publications on patients’ perceptions on choosing a treatment for SUI, we identified four main themes: “pre-existing experiences and notions that women bring to the consultations”, “aspects of treatment and patient characteristics”, “aspects of the consulting health care professional and facilities” and “ways of reaching a decision”. Before entering the consultation room, women with SUI already have ideas and expectations regarding their incontinence, the consultation and the treatment options. These notions arise from prior experiences, peer guidance and expectations on the effect of treatment. During the consultation, women reflect upon many different aspects when considering treatment options for SUI. Women mostly take into account treatment aspects such as efficacy, complications, invasiveness, recovery period and type of anaesthesia. They also regard their symptom severity, general health status, age and social roles as important factors to consider when deciding on a treatment. Cost of treatment can occasionally play a role in treatment decision making. The consulting health care professional and the facilities also have an impact on women’s decisions about treatment options, predominantly in the availability of treatment options and in the way in which the counselling is provided by the health care professional. Some women felt that the explanation of their physician was unclear and did not comprise all possible treatment options. Women use different ways of making a treatment decision. Some women choose the treatment with the highest efficacy, hoping to be cured as soon as possible. Others use a process of elimination excluding unfit options, to reach their preferred treatment, or a hierarchical approach, starting with the least invasive treatment option. The broad spectrum of considerations and viewpoints that different women take into account when reflecting on treatment options of their SUI argues against a “one treatment fits all” approach and underscores the importance of SDM in consultations with these patients.

A previous qualitative systematic review on SUI did not mention patients’ perceptions of treatment and decision making at all [18]. Another systematic review addressed women’s hindsight views of MUS counselling and reported an expertise imbalance between the physician and the patient during counselling, resulting in the patient feeling powerless [17]. This is congruent with our findings that patients want to be informed about many different treatment aspects before making a decision. However, the previous review presented perspectives of women who had already been treated making it prone to hindsight bias, whereas our study also included the moment of decision making itself. A different systematic review of some of the studies included in our scoping review only briefly mentioned that cure rates and expectations regarding treatment effects are important for women in choosing treatment for SUI, without providing more details [16].

The findings from this scoping review highlight some important current knowledge gaps in medical research. Considering that cultural background may influence the perception of incontinence symptoms and adherence to treatment [18], it is highly likely that cultural differences also have an effect on treatment decision making, but we found hardly any information on this aspect. Although we retrieved studies from multiple cultural backgrounds and countries, the influence of culture on the decision making for treatment for SUI was addressed in none, apart from the limited availability of some treatment options in a developing country [43].

In addition, only a few quotes were found on the effects of patients’ age and comorbidities on their decision making, even though it has a large impact on the treatment options that clinicians consider and offer to individual patients [42]. Just one study examined the association of personality traits on treatment perceptions as reflected by adherence [35]. Similarly, although sexuality issues play a role in women’s perception of their incontinence and the adherence to treatments such as pessary use [43, 45], little information was found on the influence of sexuality on treatment decision making for SUI. Some women related their incontinence symptoms to sexual intercourse, making it plausible that women also take this into account when making a decision on treatment [46].

The last gap we identified in the literature was the apparent lack of data on the decision-making style women prefer when making a treatment decision for their SUI. In general, patients want at least to be involved in decision making, preferring either SDM or informative decision making over paternalistic decision making by the physician alone [47]. A scoping review on SDM in surgical decisions found that female gender, higher education and younger age were associated with a patient’s preference for SDM [14]. As female SUI often starts at a relatively young age, this finding would suggest that patients with SUI would also prefer SDM. The current scoping review found some information on the way in which women with SUI make a decision, but hardly any evidence was found on the collaboration between patients and health care professionals in the decision-making process.

Although it is possible that these knowledge gaps exist because patients do not take the aforementioned aspects into account, it is more likely that these aspects were underexposed in the research methods of the studies included. To equip clinicians with better tools for counselling patients with SUI, further research is needed to understand the influences of general health status, cultural background, personality traits and sexuality on women’s views of treatment options and decision making. In addition, to provide clinicians with more insight into the optimal decision-making process in female SUI, further research is needed on patients’ preferences regarding decision-making styles for the treatment of this condition.

### Strengths and Limitations

To our knowledge, this scoping review is the first to focus on women’s perceptions of treatment decision making for SUI. Our broad search strategy resulted in the inclusion of studies from different countries and cultures on both surgical and non-surgical treatment options, ensuring a global overview of all current existing literature. This overview also allowed us to identify relevant knowledge gaps and provide possible paths for future research.

In any literature review, it is possible that relevant published studies are not found. Our sensitive search strategy included as many synonyms as possible, but other names for treatment options may exist. Although we did not apply a language limitation, our search only included English terms. By searching in three databases and checking the reference lists of all included studies, we tried to minimalise the chance of missing any relevant publications.

To allow data from different studies to be merged and avoid existing bias, we chose to recode all available data in our qualitative analysis. It is possible that the re-arrangement of codes, subthemes and themes would have shifted slightly if we had stuck to the original coding.

## Conclusions

This scoping review provides an overview of existing literature on women’s perceptions of the treatment decision-making process for SUI. Women have strong perceptions of their condition and potential treatments, which they bring to the consultation, and which play a role in the process of deciding on a treatment. Although women appreciate their physician’s advice, they want to be involved in the treatment decision to ensure that the treatment is in line with their personal views and preferences. Interestingly, however, relatively little is known about the exact issues that women take into account in this decision-making process, specifically on their background, personality traits and sexuality. Even less information is available on their preferences on the approach to decision making itself. There appears to be an urgent need for further research on these knowledge gaps to aid clinicians in the optimal counselling and decision-making approach for women with SUI.

## Supplementary Information

Below is the link to the electronic supplementary material.

Supplementary file1 (DOCX 18.0 KB)

## Data Availability

Data will be made available upon request.

## Abbreviations

MUS: Mid-urethral sling
PFMT: Pelvic floor muscle therapy
SDM: Shared decision making
SUI: Stress urinary incontinence
