# Supplementary material for: Patients’ Perceptions of Stress Urinary Incontinence Treatment: A Scoping Review of Qualitative Studies
Source: Int Urogynecol J. 2025 Feb 11;36(6):1149–62. doi: 10.1007/s00192-025-06061-w (PMC12287129; doi:10.1007/s00192-025-06061-w)
Supplement: Supplementary file 1 — Supplementary file1 (DOCX 18.0 KB) [file 192_2025_6061_MOESM1_ESM.docx]

# Appendix 1 Search Strategy 10 August 2022

*PubMed Search Strategy: 1,527 hits*

("Female"[Mesh] OR female*[tiab] OR women*[tiab] OR woman*[tiab])

AND

("Urinary Incontinence, Stress"[Mesh] OR (stress[tiab] AND urinary[tiab] AND incontinence*[tiab]) OR SUI[tiab])

AND

("Interviews as Topic"[Mesh] OR interview*[tiab] OR "Qualitative Research"[Mesh] OR “qualitative research”[tiab] OR "Clinical Decision-Making"[Mesh] OR “clinical decision making”[tiab] OR ((patient*[tiab] OR woman*[tiab] OR women*[tiab]) AND (preference*[tiab] OR perception*[tiab] OR perspective*[tiab] OR goal*[tiab] OR satisf*[tiab])))

AND

("Urogenital Surgical Procedures"[Mesh] OR “urogenital surg*”[tiab] OR "Suburethral Slings"[Mesh] OR ((“suburethral”[tiab] OR “urethral”[tiab] OR “mid-urethral”[tiab] OR “midurethral”[tiab] OR “single-incision”[tiab] OR “single incision”[tiab]) AND “sling*”[tiab]) OR ((“transobturator”[tiab] OR “suburethral”[tiab] OR “trans-obturator”[tiab] OR “trans obturator”[tiab] OR “tensionless”[tiab] OR “vaginal”[tiab] OR “tension-free”[tiab] or “tension free”) AND “tape*”[tiab]) OR “TVT”[tiab] OR “TOT”[tiab] OR “TVT-O”[tiab] OR “MUS”[tiab] OR “SIMS”[tiab] OR "Urinary Sphincter, Artificial"[Mesh] OR ((“urinary”[tiab] OR “genitourinary”[tiab]) AND “artificial”[tiab] AND “sphincter*”[tiab]) OR “urethral bulking agent*”[tiab] OR “bulking agent*”[tiab] OR "Physical Therapy Modalities"[Mesh] OR (“physical”[tiab] AND “therapy”[tiab] AND (“modalit*”[tiab] OR “technique*”[tiab])) OR “physiotherapy*”[tiab] OR (“physical”[tiab] AND “therap*”[tiab]) OR “colposuspension”[tiab] OR "Urinary Incontinence, Stress/therapy"[Mesh] OR (“urinary”[tiab] AND “incontinence”[tiab] AND “stress”[tiab] AND “therap*”[tiab]) OR "Urinary Incontinence, Stress/surgery"[Mesh] OR (“urinary”[tiab] AND “incontinence”[tiab] AND “stress”[tiab] AND “surger*”[tiab]))

*Embase Search Strategy:* *3,022 hits*

('female'/exp OR (‘female*’ OR ‘women*’ OR ‘woman*’):ab,ti,kw)

AND

('stress incontinence'/exp OR (‘stress’:ab,ti,kw AND ‘urinary’:ab,ti,kw AND ‘incontinence*’:ab,ti,kw) OR ‘SUI’:ab,ti,kw)

AND

('interview'/exp OR ‘interview*’:ab,ti,kw OR 'qualitative research'/exp OR ‘qualitative research’:ab,ti,kw OR 'clinical decision making'/exp OR ‘clinical decision making’:ab,ti,kw OR ((‘patient*’ OR ‘woman*’ OR ‘women*’):ab,ti,kw AND (‘preference*’ OR ‘perception*’ OR ‘perspective*’ OR ‘goal*’ OR ‘satisf*’):ab,ti,kw))

AND

('urologic surgery'/exp OR ‘urogenital surg*’:ab,ti,kw OR 'suburethral sling'/exp OR ((‘suburethral’ OR ‘urethral’ OR ‘mid-urethral’ OR ‘midurethral’ OR ‘single-incision’ OR ‘single incision’):ab,ti,kw AND ‘sling*’:ab,ti,kw) OR ((‘transobturator’ OR ‘suburethral’ OR ‘trans-obturator’ OR ‘trans obturator’ OR ‘tensionless’ OR ‘vaginal’ OR ‘tension-free’ or ‘tension free’):ab,ti,kw AND ‘tape*’:ab,ti,kw) OR ‘TVT’:ab,ti,kw OR ‘TOT’:ab,ti,kw OR ‘TVT-O’:ab,ti,kw OR ‘MUS’:ab,ti,kw OR ‘SIMS’:ab,ti,kw OR 'bladder sphincter prosthesis'/exp OR ((‘urinary’ OR ‘genitourinary’):ab,ti,kw AND ‘artificial’:ab,ti,kw AND ‘sphincter*’:ab,ti,kw) OR ‘urethral bulking agent*’:ab,ti,kw OR ‘bulking agent*’:ab,ti,kw OR 'physiotherapy'/exp OR (‘physical’:ab,ti,kw AND ‘therapy’:ab,ti,kw AND (‘modalit*’ OR ‘technique*’):ab,ti,kw) OR ‘physiotherapy’:ab,ti,kw OR (‘physical’ AND ‘therap*’):ab,ti,kw OR ‘colposuspension’:ab,ti,kw OR ('stress incontinence'/exp AND ‘therap*’:ab,ti,kw) OR (‘urinary’ AND ‘incontinence’ AND ‘stress’ AND ‘therap*’):ab,ti,kw OR ('stress incontinence'/exp AND ‘surger*’:ab,ti,kw) OR (‘urinary’ AND ‘incontinence’ AND ‘stress’ AND ‘surger*’):ab,ti,kw)

*CINAHL* *Search Strategy:* *166 hits*

(MH "Female" OR TI (“female*” OR “women*” OR “woman*”) OR AB (“female*” OR “women*” OR “woman*”))

AND

(MH "Stress Incontinence" OR TI (“stress” AND “urinary” AND “incontinence*”) OR AB (“stress” AND “urinary” AND “incontinence*”) OR TI “SUI” OR AB “SUI”)

AND

(MH "Interviews+" OR TI “interview*” OR AB “interview*” OR MH "Qualitative Studies+" OR TI “qualitative research” OR AB “qualitative research” OR MH "Decision Making, Clinical+" OR TI “clinical decision making” OR AB “clinical decision making” OR ((TI (“patient* OR “woman*” OR “women*”) OR AB (“patient* OR “woman*” OR “women*”)) AND (TI (“preference*” OR “perception*” OR “perspective*” OR “goal*” OR “satisf*”) OR AB (“preference*” OR “perception*” OR “perspective*” OR “goal*” OR “satisf*”)))

AND

(MH "Surgery, Urogenital+" OR TI “urogenital surg*” OR AB “urogenital surg*” OR MH "Suburethral Slings" OR ((TI (“suburethral” OR “urethral” OR “mid-urethral” OR “midurethral” OR “single-incision” OR “single incision”) OR AB (“suburethral” OR “urethral” OR “mid-urethral” OR “midurethral” OR “single-incision” OR “single incision”)) AND (TI “sling*” OR AB “sling*”)) OR ((TI (“transobturator”[tiab] OR “suburethral”[tiab] OR “trans-obturator”[tiab] OR “trans obturator”[tiab] OR “tensionless”[tiab] OR “vaginal”[tiab] OR “tension-free”[tiab] or “tension free”) OR AB (“transobturator”[tiab] OR “suburethral”[tiab] OR “trans-obturator”[tiab] OR “trans obturator”[tiab] OR “tensionless”[tiab] OR “vaginal”[tiab] OR “tension-free”[tiab] or “tension free”)) AND (TI “tape*” OR AB “tape*”)) OR TI “TVT” OR AB “TVT” TI “TOT” OR AB “TOT” OR TI “TVT-O” OR AB “TVT-O” OR TI “MUS” OR AB “MUS” OR TI “SIMS” OR AB “SIMS” OR MH "Urinary Sphincter, Artificial" OR ((TI (“urinary” OR “genitourinary”) OR AB (“urinary” OR “genitourinary”)) AND (TI “artificial” OR AB “artificial”) AND (TI “sphincter*” OR AB “sphincter*”)) OR TI “urethral bulking agent*” OR AB “urethral bulking agent*” OR TI “bulking agent*” OR AB “bulking agent*” OR (MH "Physical Therapy+") OR ((TI “physical” OR AB “physical”) AND (TI “therapy” OR AB “therapy”) AND (TI (“modalit*” OR “technique*”) OR AB (“modalit*” OR “technique*”))) OR TI “physiotherapy*” OR AB “physiotherapy*” OR ((TI “physical” OR AB “physical”) AND (TI “therap*” OR AB “therap*”)) OR TI “colposuspension” OR AB “colposuspension” OR (MH "Stress Incontinence/DH/DT/TH") OR ((TI “urinary” OR AB “urinary”) AND (TI “incontinence” OR AB “incontinence”) AND (TI “stress” OR AB “stress”) AND ( TI “therap*” OR AB “therap*”)) OR MH "Stress Incontinence/SU" OR ((TI “urinary” OR AB “urinary”) AND (TI “incontinence” OR AB “incontinence”) AND (TI “stress” OR AB “stress”) AND ( TI “surger*” OR AB “surger*”)))

# Appendix 2 Data extraction tool in Google Forms

| **Data characteristic** | **Definition** | **Extracted data** |
| --- | --- | --- |
| First author | First author on the paper | Free text |
| Publication year | Year of publication of the paper, date if available | Date |
| Country of origin | Country where the research took place | Free text |
| Setting | Care setting where the research took place | Categorical:  Primary care  Secondary care  Tertiary care |
| Type of incontinence | Type of incontinence of participants | Categorical:  SUI  MUI |
| Treatment studied | Principal treatment of SUI studied | Categorical:  Pelvic Floor Muscle Therapy (PFMT)  Colposuspension (Burch)  TVT/TOT  SIMS  Bulking agent  E-health, eg app  Not reported  Other |
| Sample size | Number of participants | Continuous |
| Age of patients | Range of age of patients | Continuous |
| Ethnicity | Ethnicity of participants | Categorical:  Asian  Black/African-American  Hispanic  White  Not reported  Other |
| Previous treatment | Treatment received before participating to the study | Categorical:  None  Pelvic Floor Muscle Therapy (PFMT)  Colposuspension (Burch)  TVT/TOT  SIMS  Bulking agent  Not reported  Other |
| Study relevance | Relevance to this scoping review | Categorical:  Patient perceptions as primary aim  Patient perceptions as secondary aim |
| Study design | The type of study design | Categorical:  Qualitative design  Mixed methods design |
| Qualitative method | The qualitative method used in the study | Categorical:  Interviews  Focus groups  Questionnaires  Other |
| Qualitative analysis method | The analysis method used in the study | Categorical:  Thematic analysis  Grounded theory  Other |
| Order of constructs | The order of constructs in the study | Categorical:  First order constructs  Second order constructs  Both |
| Themes (brief) | Overview of themes in the article | Free text |
| Themes | The in-depth themes in the article | Exact copy of text from publication |
| Strengths to research | The strengths of the research mentioned in the paper | Exact copy of text from publication |
| Limitations to research | The limitations of the research mentioned in the paper | Exact copy of text from publication |
| Recommendations to future research | The recommendations to future research mentioned in the paper | Exact copy of text from publication |
